# Disaster impacts on cost and utilization of Medicare

**DOI:** 10.1186/s12913-018-2900-9

**Published:** 2018-02-07

**Authors:** Nathanael Rosenheim, Shannon Grabich, Jennifer A. Horney

**Affiliations:** 1 Hazard Reduction and Recovery Center, 3137 TAMU, College Station, TX 77843 USA; 2 Department of Epidemiology, University of North Carolina at Chapel Hill Gillings School of Global Public Health, CB# 7435, Chapel Hill, NC 27599 USA; 3 Department of Epidemiology and Biostatistics, Texas A&M University School of Public Health, 1266 TAMU, College Station, TX 77843 USA

**Keywords:** Medicare, Aging / older adults, Access / demand / utilization, Administrative data, Determinants of health

## Abstract

**Background:**

To estimate changes in the cost and utilization of Medicare among beneficiaries over age 65 who have been impacted by a natural disaster, we merged publically available county-level Medicare claims for the years 2008–2012 with Federal Emergency Management Agency (FEMA) data related to disasters in each U.S. County from 2007 to 2012.

**Methods:**

Fixed-effects generalized linear models were used to calculate change in per capita costs standardized by region and utilization per 1000 beneficiaries at the county level. Aggregate county demographic characteristics of Medicare participants were included as predictors of change in county-level utilization and cost. FEMA data was used to determine counties that experienced no, some, high, and extreme hazard exposure. FEMA data was merged with claims data to create a balanced panel dataset from 2008 to 2012.

**Results:**

In general, both cost and utilization of Medicare services were higher in counties with more hazard exposure. However, utilization of home health services was lower in counties with more hazard exposure.

**Conclusions:**

Additional research using individual-level data is needed to address limitations and determine the impacts of the substitution of services (e.g., inpatient rehabilitation for home health) that may be occurring in disaster affected areas during the post-disaster period.

## Background

The growing U.S. adult population over age 65 remains disproportionately vulnerable to health hazards resulting from natural disasters. For instance, 64% of the people who died in Hurricane Katrina were over age 65 [36]. The frail elderly (e.g., those with impaired physical mobility, diminished sensory awareness, or chronic health conditions) and the “old old” (i.e., over age 85) are at especially high risk [18]. Several characteristics make older adults more susceptible to the effects of natural disasters, including greater hazard zone occupancy, living in less hazard-resistant structures, and lower rates of both emergency preparedness and disaster recovery responses [31]. While case studies of individual disasters can effectively characterize the impacts of a single type of disaster on health outcomes and health systems utilization in a specific geographic location, comparative data using common measurements over time and across locations are generally not available. In the absence of such knowledge, the promise of being able to proactively anticipate and mitigate the effects of future disasters on health systems utilization and cost among the U.S. population over age 65 will likely remain limited [11, 31, 33].

It has been well-established in the disaster literature that while older adults are uniquely vulnerable to disasters and typically experience greater disaster loss, they receive less social and financial support after a disaster relative to other groups. In the social context, they may hesitate to ask for assistance [3] or have fewer family members to help them get assistance [22, 23]. Older adults are also less likely to have adequate property insurance coverage and more likely to report problems with using their coverage [6, 34]. Since most persons over age 65 are eligible beneficiaries of Medicare Part A (i.e. inpatient hospital insurance that covers those over age 65, as well as younger people with certain conditions), concerns about adequate coverage and problems with using coverage to seek health care after a disaster should be mitigated to some extent by this factor. However, as Medicare beneficiaries become displaced by disasters – particularly older and disabled beneficiaries – they may experience problems with using their health insurance coverage or face higher out of network co-payments and other charges [37].

Few published research data in the U.S. have specifically examined the impact of disasters on the older adult’s cost and utilization of Medicare after disasters. In a single study after Hurricane Katrina, the rate of physician office visits among Medicare Advantage (i.e. Medicare plans offered by private companies and operated as health maintenance organizations (HMO) or preferred provider organization (PPO) plans) enrollees in four Louisiana Parishes declined by 57% compared with the mean number of visits 11 months earlier, while emergency department visits and inpatient hospitalization rates increased [7]. In a study of 213 U.S. Counties, Anderson et al. [1] used Medicare inpatient claims for fee-for-service beneficiaries over age 65 to demonstrate that respiratory hospitalizations increased 4.3% (95% CI: 3.8%, 4.8%) for each 10 °F increase in daily mean summer temperature.

Changes in the utilization of mental health services have been more widely documented among the general population and among veterans post-disaster, although findings have been mixed. After four hurricanes made landfall in Florida in 2004, veterans with posttraumatic stress disorder residing in counties affected by hurricanes utilized 28% more mental health services [19]. After Hurricane Ike, older residents of Galveston and Chambers Counties, TX, reported significant barriers (OR 1.04; 95% CI: 1.01, 1.06) to accessing mental health care, but most were preference based (i.e., they preferred to work out their own problems) [27]. Findings in the international disaster research related to the utilization of health care by older adults after a disaster are also mixed. In a cross-sectional survey following a major flood in China, Wu et al. [39] found that those over age 60 sought significantly more healthcare in the two weeks following the disaster than the older adult residents of a non-affected reference province. Following the March 2011 earthquake and tsunami in Japan, more than one-third of survivors over age 65 were considered at risk of immobility due to a lack of access to rehabilitation services [29].

Because of the case study, place-based nature of disaster research, findings are difficult to generalize to other disasters in other locations. Research focused on a single event also limits our capacity to enhance the resilience of the older adults, or the health systems that serve them, to future disasters of a different type, scale, or location. Effectively addressing the disproportionate vulnerability to the health hazards of natural disasters for older adults will require specific planning and preparation by the Medicare system. The aim of this study is to identify and quantify the impacts of disasters on costs and utilization within the Medicare system to provide insights to improve pre-disaster planning and preparedness.

## Methods

### Sample

The Centers for Medicare and Medicaid Services (CMS) Geographic Variation Public Use File (GVPUF) contains 100% of Medicare claims for the 50 U.S. States and Washington D.C., aggregated to the county level for the years 2008–2012. The GVPUF file includes demographic information on beneficiaries (e.g., age, gender, race and ethnicity), prevalence data for 19 chronic conditions, and utilization and cost data at the county level [9]. The CMS GVPUF reports county-level data for beneficiaries of all ages, including those under the age of 65 who receive Medicare (6.2 million in 2012) and those over age 65 (27.9 million in 2012) [10]. The CMS GVPUF suppresses data for counties with any data field with fewer than 11 beneficiaries; therefore, our sample included 1539 counties across the contiguous U.S. (55%) with all demographic data for all years. The final sample included 5 years of longitudinal data for each U.S. County with all demographic data, for a total sample of 7695.

### Measures

#### Exposure

County-level hazard exposure was defined using Federal Emergency Management Agency (FEMA) data related to disaster declarations for nine types of disasters, including fires, floods, hurricanes, severe ice storms, severe storms, snow, tornadoes, earthquakes, and other, as well as information on the dollar amounts distributed to counties for disaster response and recovery [14]. Disaster declarations are issued by the President of the United States under the authority of the Stafford Act (42 U.S.C. §§ 5121–5207). Dollars can be distributed in the form of federal public assistance grants to public or non-profit organizations, which may be used to replace, repair, or restore disaster-damaged publicly owned facilities [15]. Public assistance is reported at the county level. We divided aggregate public assistance dollars by the population of the county to determine public assistance per capita. Individuals could also apply for Federal assistance, which is reported for each disaster [16]. Per capita dollar amounts can be aggregated annually to account for counties that may have been exposed to multiple disasters in one year.

To account for differences in the scope and severity of different disasters over the study period, a hazard factor exposure variable was created by conducting a factor analysis using four variables available from FEMA, including: 1) the total number of days with major disaster declared, 2) the available public assistance divided by estimated county population, 3) individual housing assistance dollars approved divided by populations in all designated counties for the related disaster, and 4) other needs individual assistance dollars approved divided by populations in all designated counties for the related disaster. For analysis, the hazard exposure factor variable was divided into four categories using increments of the standard deviation. No hazard exposure was defined as counties with zero days of major disaster declaration and no public, housing, or individual assistance funds distributed (*N* = 5408). Some hazard exposure was defined as a hazard exposure factor value greater than the minimum and less than one standard deviation from the mean (*N* = 1661). High hazard exposure was defined as a hazard exposure factor value between one and two standard deviations from the mean (*N* = 282). Extremely high exposure was defined as hazard exposure factor value greater than two standard deviations from the mean (*N* = 344).

#### Outcomes

Various measures of Medicare utilization and cost included in the GVPUF were considered. Three utilization measures included days of inpatient hospital care (IP), days of care in inpatient rehabilitation facilities (IRF), and number of home health (HH) visits. All utilization measures are expressed in terms of usage per 1000 beneficiaries. Four cost measures included costs for the three utilization categories (IP, IRF, and HH) and the overall standardized per capita costs for all categories. Cost measures are standardized to account for differences in local wages and input prices which allows for evaluation across geographic areas [10].

### Analysis

FEMA disaster data was merged by County and State Federal Information Processing Standards (FIPS) code with the CMS GVPUF to create a balanced panel dataset from 2008 to 2012. Descriptive analyses were conducted to explore changes in exposures and outcomes over time. Means, standard errors, and percentages were calculated and ANOVA and Chi-Square *p*-value tests were conducted as appropriate. Changes in each of the three utilization and four cost outcome measures were described using means and standard errors for each category of the hazard exposure factor variable (e.g., no, low, high, extreme) as previously defined.

Multivariable fixed-effects models were used to evaluate the associations between hazard exposure and each utilization and cost variable. Models also included county-level demographic characteristics for Medicare participants (e.g., average age, percent female, percent African-American, percent eligible for Medicaid (i.e., health insurance for low-income persons), demographic characteristics (e.g., percent of county living below the poverty line), and year variables to control for trends. All data analyses were conducted in Stata 11 (College Station, TX) and datasets are publically available from FEMA and CMS. As these analyses use only publically available secondary data, this research was considered exempt from Institutional Review (IRB2015-0811 M).

## Results

### Hazard exposure factor

The hazard exposure factor was mean centered and ranged from − 0.33 in counties with no exposure to disaster to 20.33 in counties with the highest disaster exposure. Of 1539 counties in the sample, 19% (*N* = 293) experienced no disasters during 2007–2012, while 87% (*N* = 1409) experienced disasters of some type and severity. Counties with some exposure experienced an average of 9 days of major disaster declaration and received $6.39 in public assistance, $1.50 in housing assistance, and $0.23 in individual assistance per capita. High exposure counties experienced major disaster declarations for an average of 15 days and received per $23.22 in public assistance, $9.48 in housing assistance, and $1.65 in individual assistance per capita. Extreme exposure counties experienced an average of 26 days of major disaster declaration and received $50.87 of public assistance, $14.21 in housing assistance, and $3.24 in individual assistance per capita.

Counties categorized with extreme hazard exposure were located in every region of the country except the Pacific Northwest and California. Due to major hurricane events in 2008, extreme hazard exposure counties were clustered in Texas (*N* = 48) and Louisiana (*N* = 47). The mid- and north-Atlantic states also had a cluster of extreme hazard exposure counties (*N* = 64) due to the August 2011 earthquake near Washington D.C., Hurricane Irene in November 2011, and Hurricane Sandy in October 2012. Extreme hazard exposure was also related to fire, flooding, earthquake, and severe storm events. Clusters of counties in Indiana were affected by severe winter storms in 2008 (*N* = 21), while clusters of counties in Texas were impacted by wildfires in 2011 (*N* = 57). Severe spring storms in Alabama in 2011 (*N* = 37) and the landfall of Hurricane Irene in North Carolina in 2011 (*N* = 26) accounted for additional clusters of extreme exposure. Counties with some or no hazard exposure were more evenly distributed across the United States and across all five years in the study. The mean and standard error of each independent variable by exposure category is presented in Table 1.

**Table 1:** Mean and standard deviation of independent variables by exposure category

	No Hazard Exposure *N* = 5408 (70.3%)	Some Hazard Exposure *N* = 1661 (21.6%)	High Hazard Exposure *N* = 282 (3.7%)	Extreme Hazard Exposure *N* = 344 (4.5%)	Total *N* = 7695
IP Covered Days Per 1000 Beneficiaries	1649.58 (369.35)	1704.03 (351.34)	1718.15 (333.95)	1863.73 (347.20)	1673.86 (366.37)
IP Per User Standardized Costs	13,614.92 (1027.47)	13,576.71 (1058.03)	13,541.02 (918.33)	13,732.44 (1094.56)	13,609.25 (1033.89)
IRF Covered Days Per 1000 Beneficiaries	133.60 (94.22)	138.39 (95.31)	158.98 (100.31)	173.16 (104.97)	137.41 (95.65)
IRF Per User Standardized Costs	17,786.85 (2086.66)	17,742.14 (2121.81)	18,075.64 (2133.70)	17,861.83 (2449.86)	17,791.31 (2114.56)
HH Visits Per 1000 Beneficiaries	3239.62 (2896.64)	3161.25 (3183.37)	3584.90 (3031.35)	4734.87 (3188.24)	3304.21 (2997.07)
HH Per User Standardized Costs	5384.45 (1793.77)	5231.35 (1704.37)	5621.85 (1932.28)	6390.39 (2154.04)	5406.22 (1812.71)
Standardized per capita costs	8579.95 (1335.85)	8553.46 (1171.36)	8909.16 (1321.37)	9274.74 (1426.68)	8618.41 (1314.89)
Count of beneficiaries	18,997.76 (31,773.16)	22,156.37 (40,297.99)	18,148.95 (21,702.84)	19,422.10 (41,966.08)	19,674.11 (34,047.59)
Average age of beneficiary	70.92 (1.51)	71.03 (1.55)	71.08 (1.52)	70.67 (1.78)	70.94 (1.53)
Female, %	54.88 (2.06)	55.14 (1.94)	55.65 (1.82)	54.80 (2.33)	54.96 (2.05)
African American, %	8.46 (10.84)	7.81 (10.36)	10.87 (13.10)	11.93 (11.62)	8.57 (10.90)
Eligible for Medicaid, %	21.20 (7.62)	21.11 (7.73)	20.58 (7.58)	23.63 (8.04)	21.27 (7.68)
People below poverty line, %	16.23 (5.59)	15.46 (5.59)	16.31 (6.32)	16.83 (5.52)	16.09 (5.63)

mean coefficients; sd in parentheses

Source: CMS GVPUF

### Descriptive analyses

There were no substantive differences in the population demographic characteristics of the study counties over the five year period. For the 1539 U.S. counties in the sample, the average age of beneficiaries was approximately 71 years, 55% of the sample was female, 8.25% was African-American, and 20.7% were eligible for both Medicare and Medicaid. Between 2008 and 2012, the average age and percent female decreased slightly, while the percent eligible for Medicaid increased less than 1%. The percent of people below poverty increased more than 2% between 2008 and 2012.

There were demographic differences between counties by hazard exposure level. Counties with extreme hazard exposure had populations with a larger percentage of African-Americans and larger percentages of people eligible for Medicaid and living below poverty (Table 2). These findings are consistent with an extensive literature on higher hazard zone occupancy by socially vulnerable populations [5, 12, 32].

**Table 2:** One-Way ANOVA Regression Coefficients for Demographic Variables by Hazard Exposure Level

	No Hazard Exposure *N* = 5408 (70.3%)	Some Hazard Exposure *N* = 1661 (21.6%)	High Hazard Exposure *N* = 282 (3.7%)	Extreme Hazard Exposure *N* = 344 (4.5%)	Constant
Average age of beneficiary	0	0.30^***^	0.83^***^	−0.26^**^	70.70^***^
Female, %	0	0.54^***^	1.19^***^	−0.29^**^	54.49^***^
African American, %	0	−1.35^***^	−0.79	8.23^***^	8.96^***^
Eligible for Medicaid, %	0	0.22	−0.1	4.05^***^	20.90^***^
People below poverty line, %	0	−0.71^***^	−1.45^***^	1.46^***^	16.61^***^

Source: CMS GVPUF and FEMA

^**^p < 0.01, ^***^p < 0.001

Counties with higher levels of hazard exposure generally had higher mean values for all categories of Medicare utilization and cost (Table 3). Mean values for all cost and utilization outcomes were lowest in counties with no hazard exposure, except IRF standardized per capita costs. These differences suggest a positive correlation between hazard exposure and Medicare utilization and costs that was further explored via multivariable modeling.

**Table 3:** Mean, Standard Deviation and One-Way ANOVA Regression Coefficients for County-Level Medicare Utilization and Cost by Hazard Exposure Level

	No Hazard Exposure *N* = 5408 (70.3%)	Some Hazard Exposure *N* = 1661 (21.6%)	High Hazard Exposure *N* = 282 (3.7%)	Extreme Hazard Exposure *N* = 344 (4.5%)	Constant
IP Covered Days Per 1000 Beneficiaries	0	90.85^***^	281.87^***^	371.38^***^	1564.46^***^
IP Per User Standardized Costs	0	37.65	338.76^***^	430.08^***^	13,528.44^***^
IRF Covered Days Per 1000 Beneficiaries	0	12.50^***^	17.67^***^	91.87^***^	122.05^***^
IRF Per User Standardized Costs	0	92.25	−447.18^***^	92.5	17,754.18^***^
HH Visits Per 1000 Beneficiaries	0	697.21^***^	775.02^***^	4455.27^***^	2469.43^***^
HH Per User Standardized Costs	0	362.97^***^	338.68^***^	3025.46^***^	4925.35^***^
Standardized per capita costs	0	323.16^***^	675.22^***^	2139.16^***^	8195.54^***^
Count of beneficiaries	0	− 396	16,479.69^***^	− 4335.83^*^	18,481.80^***^

Source: CMS GVPUF and FEMA

^*^p < 0.05,^***^p < 0.001

### Multivariable modeling

#### Medicare utilization

Overall, fixed-effects models demonstrate that the utilization of Medicare services at the county level are impacted by natural hazards. As hazard exposure increases, services such as HH are negatively impacted. Table 4 summarizes the results for the fixed-effects models for Medicare utilization, including the number of covered days for IP and IRF per 1000 beneficiaries and the number of HH visits per 1000 beneficiaries. Counties with no hazard exposure in 2007 or 2008 are represented in the constant.

**Table 4:** Fixed-Effects Model Estimates for Medicare Utilization

	IP Covered Days Per 1000 Beneficiaries		IRF Covered Days Per 1000 Beneficiaries		HH Visits Per 1000 Beneficiaries	
	β	95% CI	β	95% CI	β	95% CI
Average age of beneficiary	19.00^***^	11.38,26.62	0.05	−1.98,2.08	65.42^*^	1.71,129.14
Female, %	7.13	−0.15,14.41	−0.66	−2.63,1.30	−42.35	−103.35,18.65
African American, %	4.95	−1.15,11.04	−0.05	−1.65,1.56	50.40	−0.55,101.36
Eligible for Medicaid, %	20.80^***^	18.49,23.11	0.61	−0.01,1.22	30.42^**^	11.09,49.75
People below poverty line, %	0.58	−1.16,2.33	−0.15	−0.61,0.31	−3.31	−17.91,11.29
2009	−94.16^***^	−101.24,-87.08	0.48	−1.40,2.35	246.53^***^	187.33,305.73
2010	− 141.42^***^	− 149.29,-133.56	−3.37^**^	−5.45,-1.28	283.53^***^	217.72,349.34
2011	−195.97^***^	− 205.01,-186.93	−3.93^**^	−6.33,-1.53	91.86^*^	16.21,167.50
2012	−274.42^***^	− 284.06,-264.78	−5.92^***^	−8.48,-3.37	−50.86	−131.58,29.85
*Hazard Exposure*						
Some	−1.95	−8.08,4.18	1.48	−0.14,3.11	36.73	−14.50,87.96
High	−17.36^**^	−30.54,-4.18	4.90^**^	1.44,8.36	0.47	−109.72,110.65
Extreme	−1.48	−13.74,10.77	0.19	−3.00,3.39	−131.73^*^	− 234.20,-29.26
*Previous year Hazard Exposure*						
Some	0.74	−5.26,6.74	0.02	−1.56,1.61	34.07	−16.10,84.23
High	−18.15^**^	−30.64,-5.66	3.48^*^	0.21,6.75	−40.13	− 144.54,64.28
Extreme	16.05^*^	2.91,29.19	−2.02	−5.46,1.41	30.88	−78.99,140.75
Constant	− 423.82	− 1047.32,199.68	162.17	−3.45,327.79	− 194.18	− 5410.22,5021.86
Observations	7695		7387		7689	
*R* ^2^	0.553		0.013		0.042	

Source: CMS GVPUF and FEMA

^*^p < 0.05, ^**^p < 0.01, ^***^p < 0.001

The number of IP covered days decreased 16.4% between 2008 and 2012, with 275 fewer covered days in 2012 when compared to 2008. High hazard exposure in both the current and the previous year had a significant negative effect on IP covered days. However, in counties with extreme hazard exposure in the previous year, IP covered days increased. IP covered days also increased as the average age of a county’s Medicare beneficiaries increased and when the percentage of Medicaid eligible participants increased.

Utilization of IRF was small compared to IP and HH, with an average of 140 days of IRF per 1000 beneficiaries compared to over 3000 HH visits per 1000 beneficiaries. High hazard exposure had a positive effect on IRF covered days. There were no significant changes in IRF covered days associated with the demographic characteristics of county Medicare beneficiaries.

In general, Medicare-covered HH visits per 1000 beneficiaries have been increasing over time, with increases of 7.5% in 2009 and 2010. HH utilization at the county-level also increased as the average age and the percent of Medicaid eligible beneficiaries increased. Even with these positive overall trends in the use of HH, utilization of HH per 1000 beneficiaries was significantly reduced by extreme hazard exposure. In years where a county experienced extreme hazard exposure, HH visits decline by 4%, or approximately 131 visits per 1000 beneficiaries.

#### Medicare cost

In general, all standardized Medicare costs per capita increased over the study period. Standardized costs per user, which include all possible Medicare related costs, increased significantly each year between 2008 and 2012, as expected given overall trends in healthcare expenditures. Some hazard exposure and extreme hazard exposure in the current year had a significant and negative effect on overall standardized annual per capita costs, while prior year exposure had no effect. Increases in average beneficiary age, percent African-American, and percent eligible for Medicaid were also significantly associated with increased overall standardized costs. However, an increase in the percentage of female beneficiaries was associated with reductions in standardized per capita costs. Table 5 summarizes the results for the fixed-effects model of standardized Medicare costs for IP, IRF, HH, and overall costs per Medicare beneficiary. Counties with no hazard exposure in 2007 or 2008 are represented in the constant.

**Table 5:** Fixed-Effects Model Estimates for Medicare Standardized Costs

	IP Per User Annual Standardized Costs		IRF Per User Annual Standardized Costs		HH Per User Annual Standardized Costs		Standardized Annual Per Capita Costs	
	β	95% CI	β	95% CI	β	95% CI	β	95% CI
Average age of beneficiary	31.57	−17.01,80.15	−67.55	−197.87,62.76	− 1.22	−32.28,29.85	82.16^***^	58.53,105.79
Female, %	−100.46^***^	−146.86,-54.06	20.79	−105.09,146.66	−9.70	−39.45,20.04	−27.24^*^	−49.80,-4.67
African American, %	80.62^***^	41.77,119.47	42.78	−60.03,145.59	−9.37	−34.21,15.48	57.84^***^	38.95,76.74
Eligible for Medicaid, %	1.85	−12.88,16.59	27.30	−11.97,66.57	25.23^***^	15.80,34.65	53.14^***^	45.98,60.31
People below poverty line, %	−8.72	−19.85,2.41	−12.41	−42.07,17.25	−3.09	− 10.21,4.03	3.17	−2.24,8.59
2009	584.15^***^	539.03,629.28	430.54^***^	310.39,550.68	423.87^***^	395.01,452.74	368.24^***^	346.29,390.18
2010	729.90^***^	679.77,780.03	1166.64^***^	1033.08,1300.21	574.67^***^	542.58,606.76	571.64^***^	547.26,596.02
2011	684.15^***^	626.53,741.78	1494.11^***^	1340.16,1648.07	313.45^***^	276.57,350.34	543.93^***^	515.90,571.95
2012	1002.25^***^	940.82,1063.69	1914.05^***^	1750.19,2077.91	210.58^***^	171.22,249.94	550.58^***^	520.70,580.45
*Hazard Exposure*								
Some	−122.04^***^	−161.10,-82.97	52.72	−51.53,156.98	−0.48	−25.46,24.50	−38.48^***^	−57.48,-19.48
High	55.07	−28.95,139.09	253.39^*^	31.61,475.17	−12.78	−66.51,40.95	12.62	−28.24,53.49
Extreme	−23.08	−101.22,55.06	389.95^***^	185.07,594.83	−18.74	−68.70,31.23	−78.23^***^	− 116.24,-40.23
*Previous year Hazard Exposure*								
Some	−106.59^***^	− 144.84,-68.33	112.00^*^	10.04,213.96	29.39^*^	4.92,53.85	−16.81	−35.42,1.79
High	−0.49	−80.10,79.13	252.15^*^	42.38,461.92	−61.07^*^	− 111.98,-10.15	18.88	−19.84,57.60
Extreme	55.55	−28.23,139.33	283.56^*^	63.04,504.08	47.42	−6.15,101.00	36.34	−4.40,77.09
Constant	15,745.10^***^	11,770.43,19,719.76	19,606.12^***^	8978.26,30,233.98	5281.16^***^	2737.67,7824.65	2190.86^*^	257.78,4123.94
Observations	7695		7387		7689		7695	
*R* ^2^	0.316		0.214		0.259		0.482	

Source: CMS GVPUF and FEMA

^*^p < 0.05, ^**^p < 0.01, ^***^p < 0.001

Annual, standardized per capita IP costs increased significantly each year between 2008 and 2012. Controlling for county-level demographic characteristics and time trends, some hazard exposure in both the current and the previous year had a negative effect on IP costs. Although the effect is small, it is similar in magnitude to cost reductions associated with an increase in the percent of female beneficiaries. Counties that experienced high and extreme hazard exposure did not have a significant increase in IP costs.

Annual, standardized per capita IRF costs also increased significantly each year between 2008 and 2012. A consistent trend was seen between increased hazard exposure and IRF standardized costs. Controlling for county-level demographic characteristics and time trends, both high and extreme levels of hazard exposure in the current year, and all levels of hazard exposure levels in the previous year, had positive effects on IRF costs. When hazard exposure is high, IRF standardized costs could increase as much as $1085 per user in years with a disaster, and as much as $786 per user the year after a disaster. Counties that experience extreme hazard exposure could see an increase in IRF costs between 1% and 3%. None of the county-level demographic characteristics had a significant effect on IRF per capita standardized costs.

Standardized per capita HH costs also increased significantly each year between 2008 and 2012. Current year hazard exposure did not have a significant effect on HH costs. However, previous year hazard exposure did. Some hazard exposure in the previous year was associated with an increase in HH costs, while high exposure in the previous year was associated with a reduction in HH costs. Among the demographic characteristics, only an increase in the percent eligible for Medicaid in a county was associated with an increase in standardized per capita HH costs.

## Discussion

Effectively addressing older adult’s disproportionate vulnerability to the health hazards of natural disasters requires specific planning and preparation by Medicare, the health insurance system that predominately serves them. The ability to accurately quantify the impacts of different types of disasters, in different locations, and at different points in time on costs and utilization within the Medicare system may provide insights to improve pre-disaster planning and preparedness, as well as post-disaster response. To quantify the health impacts of natural disasters on older adults in the U.S., we conducted a study of publically available, county-level Medicare claims to determine the impact of disaster exposure on utilization of Medicare and the costs associated with this utilization.

Based on a large literature demonstrating that older adults suffer disproportionately from disasters, and are more likely than younger people to experience morbidity, mortality, or other health impacts as the result of disasters, we hypothesized that Medicare utilization and costs would increase in counties impacted by a disaster, and that these increases would be larger in counties more severely impacted by disaster [2, 4, 8, 17, 21, 24–26, 30, 38]. We found some evidence to support the hypothesis that cost and utilization would increase at the county level in years with more severe hazard exposure. Current year high hazard exposure was associated with significant increases in IRF covered days per 1000 beneficiaries as well as higher IRF per capita standardized costs. However, we also found an inverse relationship between IRF and HH utilization in areas exposed to higher hazard levels. Extreme hazard exposure was negatively associated with HH visits per 1000 beneficiaries and any disaster exposure was associated with reductions in HH standardized costs. This relationship suggests further investigation is needed into the whether IRF may be substituted for HH in cases where beneficiaries evacuated or their HH provider was unable to reach them due to the impacts of the disaster.

Much of the published research on the impacts of disasters on health categorizes exposure based on a dichotomous variable of whether or not a county had a major disaster declaration from FEMA or on spatial data related to storm trajectories [28, 35]. By creating the hazard exposure factor variable, which incorporates additional publically available FEMA data related to the number of days the disaster declaration was in place and the total spending for public, individual, and housing assistance, we were able to account for additional information about the size, scope, and potential impact of the disasters. This new hazard factor exposure variable is easily reproducible from public data, and helps to clarify the interpretation of correlations between hazard exposure intensity and Medicare utilization and costs within the fixed effects model.

This study has several important limitations. Because the CMS GVPUF file suppresses data for any field with fewer than 11 observations, many rural counties were not included in the final study sample. As older residents of rural areas are likely to face unique concerns with regard to access to care during both the disaster and inter-disaster periods, this study may not represent those beneficiaries’ experiences well. However, the suppressed counties include only 5% of the total U.S. population. In addition, accurate estimation of the effects of disasters on utilization and cost may be limited by the use of county-level data that can create potential exposure misclassification. Beneficiaries may have lived in a part of the county that was not impacted by the disaster or may have evacuated prior to the disaster and sought health care elsewhere or from other providers. However, this potential for exposure misclassification is a problem in most disaster research, since FEMA disaster declarations are issued at the county level, which is not likely to be the best spatial level at which to characterize exposures [20]. This type of analysis may also have resulted in an underestimation of effects since beneficiaries who die are removed from the dataset, particularly if those exposed to disaster have higher death rates that those who were not exposed [13]. Assocations could have been confounded by unmeasured confounders, which could have biased our results. This analysis only addresses claims from a single health insurance system in the U.S., Medicare, which is primarily utilized by the elderly. We cannot therefore generalize our findings to other health insurance networks. The research team has planned a follow-up study using restricted access individual-level data from the National Center for Health Statistics (NCHS). The NCHS-CMS Medicare linked data file contains individual-level Medicare claims data from 1999 to 2007 for all individuals that participated in the National Health Interview Survey between 1997 and 2005.

## Conclusions

Exposure to all types of natural disasters, including fires, floods, hurricanes, severe ice storms, severe storms, snow, tornadoes, and earthquakes can have an important effect on the utilization and cost of health care for older adults in the U.S. Although these findings may not be immediately translatable to actions that local area agencies can take to prepare and protect their residents, it does give additional insight into the impacts of disaster on the health and health system utilization of the older adults after different types of disasters. Over time, these findings, as well as additional planned analyses of both publically available and restricted access data, should help inform the development of evidence-based strategies (e.g., improvements to hospital or health system preparedness plans and development of pre-disaster interventions for chronically ill older adults) to mitigate the impact of future disasters on this vulnerable population and improve disaster resilience among individuals covered by Medicare.

## Abbreviations

CMS: Centers for Medicare and Medicaid Services
FEMA: Federal Emergency Management Agency
GVPUF: Geographic Variation Public Use File
HH: Home Health
IP: Inpatient Care
IRF: Inpatient Rehabilitation Facilities
NCHS: National Center for Health Statistics

## References

[1] Anderson GB, Dominici F, Wang Y, McCormack MC, Bell ML, Peng RD (2013). Heat-related emergency hospitalizations for respiratory diseases in the Medicare population. Am J Respir Crit Care Med.

[2] Beaglehole B (2011). Christchurch earthquake. Aust N Z J Psychiatry.

[3] Bell BD (1978). Disaster impact and response: overcoming the thousand natural shocks. Gerontologist.

[4] Berggren RE, Curiel TJ (2006). After the storm -- health care infrastructure in post-Katrina new Orleans. N Engl J Med.

[5] Blaikie P, Cannon T, Davis I, Wisner B (2014). At risk: natural hazards, people's vulnerability and disasters.

[6] Burnett J, Dyer CB, Pickins S (2007). Rapid needs assessments for older adults in disasters. Generations.

[7] Burton LC, Skinner EA, Uscher-Pines L, Lieberman R, Leff B, Clark R, Yu Q, Lemke KW, Weiner JP (2009). Health of Medicare advantage plan enrollees at 1 year after hurricane Katrina. Am J Manag Care.

[8] Cefalu WT, Smith SR, Blonde L, Fonseca V (2006). The hurricane Katrina aftermath and its impact on diabetes care: observations from "ground zero": lessons in disaster preparedness of people with diabetes. Diabetes Care.

[9] Centers for Medicare and Medicaid Services (2013). Public Use File New Data on Geographic Variation.

[10] Centers for Medicare and Medicaid Services (2013). Medicare data for the geographic variation public use file: a methodological overview.

[11] Cutter SL, Emrich CT (2006). Moral hazard, social catastrophe: the changing face of vulnerability along the hurricane coasts. Ann Am Acad Polit. Soc Sci.

[12] Cutter SL, Emrich CT, Webb JJ, Morath D. Social vulnerability to climate variability hazards: a review of the literature: Final Report to Oxfam America; 2009. Available at: http://citeseerx.ist.psu.edu/viewdoc/summary?doi=10.1.1.458.7614. Accessed 02 Feb 2018.

[13] Dosa D, Feng Z, Hyer K, Brown LM, Thomas K, Mor V (2010). Effects of hurricane Katrina on nursing resident mortality, hospitalization, and functional decline. Disaster Med Public Health Prep..

[14] Federal Emergency Management Agency (2014). FEMA disaster declarations summary - open government dataset.

[15] Federal Emergency Management Agency (2014). FEMA Public Assistance Funded Projects Detail - Open Government Initiative.

[16] Federal Emergency Management Agency. 2014c. “FEMA Disaster Declarations by Year” [Accessed 8 July 2014]. Available at: https://www.fema.gov/disasters/year.

[17] Ferdinand KC (2005). The hurricane Katrina disaster: focus on the hypertensive patient. J Clin Hypertens.

[18] Fernandez LS, Byard D, Lin CC, Benson S, Frail BJA (2002). Elderly as disaster victims: emergency management strategies. Prehosp Disaster Med.

[19] Frahm KA, Barnett SD, Brown LM, Hickling EJ, Olney R, Campbell RR, Lapcevic WA (2013). Posttraumatic stress disorder and use of psychiatric and alcohol related services: the effect of the 2004–2005 Florida hurricane seasons on veterans. Community Ment Health J.

[20] Grabich SC, Horney JA, Konrad C, Measuring LDT (2015). The storm: methods of quantifyng hurricane exposure with pregnancy outcomes. Nat Hazards Rev.

[21] Kessler RC, Hurricane Katrina Community Advisory Group (2007). Hurricane Katrina's impact on the care of survivors with chronic medical conditions. J Gen Intern Med.

[22] Kaniasty K, Norris F (1995). In search of altruistic community: patterns of social support mobilization following hurricane Hugo. Am J Community Psychol.

[23] Kilijanek TS, Drabek TE (1979). Assessing long-term impacts of a natural disaster: a focus on the elderly. Gerontologist.

[24] Kleinpeter MA, Norman LD, Krane NK (2006). Dialysis services in the hurricane-affected areas in 2005: lessons learned. Am J Med Sci.

[25] Knowles RM, Garrison B (2006). Planning for elderly in natural disasters. Disaster Recovery Journal.

[26] Krousel-Wood MA, Islam T, Muntner P, Stanley E, Phillips A, Webber LS, Frohlich ED, Re RN (2008). Medication adherence in older clinic patients with hypertension after hurricane Katrina: implications for clinical practice and disaster management. Am J Med Sci.

[27] Lowe SR, Joshi S, Pietrzak RH, Galea S, Cerdá M (2015). Mental health and general wellness in the aftermath of hurricane Ike. Soc Sci Med.

[28] McCarthy FX. FEMA’s disaster declaration process: A primer [Accessed 19 July 2016]. Available at: http://www.fas.org/sgp/crs/homesec/RL34146.pdf.

[29] Michiaki T (2012). Rehabilitation support in the Kesen-Numa earthquake and tsunami disaster area. Japanese J Rehabil Med.

[30] Mokdad AH, Mensah GA, Posner SF, Reed E, Simoes EJ, Engelgau MM (2005). When chronic conditions become acute: prevention and control of chronic diseases and adverse health outcomes during natural disasters. Prev Chronic Dis.

[31] National Research Council (2006). Hazards and disaster research, social science, and public policy.

[32] Peacock WG, Grover H, Mayunga J, Van Zandt S, Brody SD, Kim HJ, Center R (2011). The status and trends of population social vulnerabilities along the Texas coast with special attention to the coastal management zone and hurricane Ike: the coastal planning atlas and social vulnerability mapping tools.

[33] Peacock WG, Kunreuther H, Hooke WH, Cutter SL, Chang SE, Berke PR (2008). Toward a resiliency and vulnerability observatory network: RAVON.

[34] Perry RW, Lindell MK (1997). Aged citizens in the warning phase of disasters: re-examining the evidence. Int J Aging Hum Dev.

[35] Powell MD, Houston SH, Amat LR, Morisseau-Leroy N (1998). The HRD real-time hurricane wind analysis system. J Wind Eng Ind Aerodyn.

[36] Stephens KU, Grew D, Chin K, Kadetz P, Greenough PG, Burkle FM, Robinson SL, Excess FER (2007). Mortality in the aftermath of hurricane Katrina: a preliminary report. Disaster Med Public Health Prep.

[37] Super N, Biles B. Displaced by hurricane Katrina: issues and options for Medicare beneficiaries. Henry J. Kaiser Family Foundation Medicare policy brief. Retrieved from: http://www.kff.org/medicare/7437.cfm. Published October 31, 2005. Accessed 6 Apr 2015.

[38] Wang PS, Gruber MJ, Powers RE, Schoenbaum M, Speier AH, Wells KB, Kessler RC (2007). Mental health service use among hurricane Katrina survivors in the eight months after the disaster. Psychiatr Serv.

[39] Wu J, Xiao J, Li T, Li X, Sun H, Chow E, Lu Y, Tian T, Li X, Wang Q, Zhuang Z, Zhang L (2015). A cross-sectional survey on the health status and the health-related quality of life of the elderly after flood disaster in Bazhong city, Sichuan, China. BMC Public Health.

